# Release principle of peptides and amino acids during the autolysis of shrimp head from *Litopenaeus vannamei* after UV‐C irradiation stress

**DOI:** 10.1002/fsn3.1288

**Published:** 2019-12-02

**Authors:** Wenhong Cao, Shen Tian, He Wang, Chaohua Zhang, Jianjun Yuan

**Affiliations:** ^1^ College of Food Science and Technology Guangdong Ocean University Zhanjiang China; ^2^ Guangdong Provincial Key Laboratory of Aquatic Product Processing and Safety Zhanjiang China; ^3^ Province Engineering Laboratory for Marine Biological Products Zhanjiang China; ^4^ College of Oceanology and Food Science Quanzhou Normal University Quanzhou China; ^5^ Fujian Province Key Laboratory for the Development of Bioactive Material from Marine Algae Quanzhou China

**Keywords:** autolysis, kinetics, *Litopenaeus vannamei*, peptides release, shrimp head

## Abstract

UV‐C irradiation can activate endogenous enzymes in the body of many aquatic animals. Autolysis kinetics of shrimp head after UV‐C irradiation stress was investigated. During the first 5 hr of autolysis, the release of the autolysis products was in line with the first‐order equations of the reaction rate: *Y* = 37.681e^−0.173^ *^t^*, *Pe* = −1.769*Y* + 74.156, and *TP* = −1.5117*Y* + 60.866. A good linear correlation was founded between the release of total protein and that of products with molecular weight of 3,000 Da after these products associated with residual total protein were autolyzed. In contrast to the inconsistent effect of substrate concentration on autolysis rate constants, the effects of pH and temperature on the autolysis rate constants of shrimp head showed a consistent pattern. An Arrhenius equation (ln*Ka = *8,090.2/*T* − 26.497) was established to validate the proposed autolysis kinetic equations. The autolysis rate of products with molecular weight < 3,000 Da increased rapidly from 0 hr to 3 hr. The aliphatic amino acids showed a higher amount release than that of other amino acids during the autolysis. The amounts of released heterocyclic amino acids (Trp and His) were also much more than that of other amino acids.

## Graphical abstract

The principle of peptides and amino acid release during the autolysis of shrimp head after UV‐C irradiation stress were investigated. The release kinetics equation of nitrogen‐containing substances in the autolysis process of shrimp head within 0–5 hr can be expressed with first‐order reaction rate equations. The results will provide important reference for high value recovery and utilization of shrimp‐head protein.

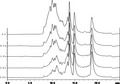

## 1 INTRODUCTION

Chinese fishery has undergone remarkable development in the past 20 years. For example, national shrimp production through marine and freshwater culture alone reached approximately 3,500,000 t in 2017 (Ministry of Agriculture & Rural Areas of China, 2018). Most of the shrimp produced in China are mainly processed as peeled and headless shrimp for export. Shrimp processing results in the disposal and discharge of more than 500,000 t of shrimp‐head waste each year. The decomposition and decay of these wastes in the absence of appropriate disposal measures result in serious environmental hazards. Shrimp‐head waste is a rich source of chitin (Sjaifullah & Santoso, 2016), proteins, peptides, nutritive components (Cahú et al., 2012; Cao et al., 2009; Yuan, Li, Pan, Wang, & Chen, 2018), and enzymes (Heu, Kim, Shahidi, & Jeong, 2003; Shahidi & Synowiecki, 1991). Previous studies have proven that hydrolysates derived from shrimp‐waste autolysis have high biological value. For example, a study showed that the biological responses of rats fed with shrimp‐waste hydrolysate were greater than those of rats fed with the casein control diet. This result indicates that shrimp‐waste hydrolysate is a potential human diet supplement (Silva et al., 2017). Therefore, the utilization of shrimp‐head waste has drawn considerable research interest in recent years. The shrimp is a special aquatic animal, and its digestive organs are mainly located in its head. Shrimp heads are rich in various endogenous enzymes, including proteases. Under certain conditions, endogenous enzymes in the shrimp body can be activated, and autolysis occurs in corresponding tissues, which function as biological macromolecular substrates. The autolysis of tissues generates various products with low molecular weight (MW). Proteins in biological tissue are mainly released in the form of soluble low‐MW peptides and amino acids under the action of endogenous enzymes. Autolysis is an effective, low‐cost, and pollution‐free method for protein recovery that does not require the use of expensive exogenous enzymes. Furthermore, numerous low‐MW peptides derived from food proteins exhibit certain bioactivities, such as antioxidant, antihypertensive, immunoregulatory, and anticancer activities (Ambigaipalan & Shahidi, 2017; Chalamaiah, Yu, & Wu, 2018; Erdmann, Grosser, & Schipporeit, 2006). Shrimp‐head autolysis is an excellent putative approach for the preparation for bioactive peptides. Autolytic efficiency depends on endogenous enzyme activity. However, the rate of spontaneous autolysis is slow.

Ultraviolet (UV) radiation is a nonthermal, low‐cost, and environmentally friendly technology that has been successfully applied in food preservation and decontamination (Chun, Kim, Lee, Yu, & Song, 2010). The increment in the gel strength of fish surimi after UV irradiation may be attributed to UV irradiation‐induced changes in the spatial structure of fish protein (Bhat & Karim, 2009); it may be due to the UV irradiation‐induced changes in the spatial structure of fish protein. Kristo, Hazizaj, and Corredig (2012) observed that UV‐induced changes in protein conformation enhanced the susceptibility of whey proteins to pepsin hydrolysis. Our research team found that UV irradiation enhanced the rate of shrimp tissue autolysis. Cao, Tan, Zhan, Li, and Zhang (2014) investigated a novel autolysis method that involved the application of C‐band UV irradiation and gradient temperature to recover protein from the heads of Litopenaeus vannamei. Proteolytic activity in shrimp heads subjected to 30 W of UV irradiation at 253.7 nm for 20 min increased by 62% relative to that in the untreated samples. Nevertheless, the principle underlying the release of proteins from shrimp heads after UV irradiation stress remains unclear.

The present study aimed to establish autolysis kinetic models, which can evaluate the influence of temperature, pH, and substrate concentration on the autolysis of *Litopenaeus vannamei* shrimp head and describe the peptides release principle of shrimp head after UV irradiation stress. The results could be expected to provide important reference for protein recovery and bioactive peptides preparation from shrimp head assisted by a physical means.

## 2 MATERIALS AND METHODS

### 2.1 Materials

Live shrimp (*L. vannamei*) (20.3 ± 0.7 g in weight, 12.2 ± 0.3 cm in length, and about three months old) were obtained from the local aquaculture base of Zhanjiang Guolian Fisheries Co., Ltd. The shrimp were transported to the laboratory in oxygen flushing seawater tank within approximately 1 hr. Upon arrival, the shrimp were washed with clean water and beheaded and the heads were collected. The collected shrimp heads stored in an ultralow temperature freezer at −75 ℃ prior to use.

MW standards (e.g., triosephosphate isomerase [26,625 Da], myoglobin [16,950 Da], α‐lactalbumin [14,437 Da], aprotinin [6,512 Da], oxidized insulin b chain [3,496 Da]), and bacitracin (1,423 Da) were obtained from Bio‐Rad. N‐Hippuryl‐His‐Leuhydrate (429.47 Da) was procured from Sigma‐Aldrich.

### 2.2 Experimental methods

#### 2.2.1 UV‐C irradiation stress

Shrimp heads were homogenized in a blender (DS‐1, Precision Instruments Co., Ltd) without water. The shrimp‐head homogenate was subjected to UV‐C irradiation in accordance with the method of Cao et al. (2014). The proteolytic activity calculated for shrimp heads subjected to UV‐C irradiation was 525 U/g shrimp head. The homogenate (100 g) was thinly spread on sterile oven dishes (diameter, 10 cm) and exposed to a UV light source (253.7 nm, 30 W, Cnlight Co., Ltd) placed at a height of 20 cm for 20 min. The treated samples were transferred aseptically to wide‐mouthed bottles for further experiments.

#### 2.2.2 Autolysis of shrimp head after UV‐C irradiation stress

Shrimp heads were autolyzed in accordance with the method used by Cao et al. (2009) with some modifications. The shrimp‐head homogenate (30 g) was poured into a conical bottle after UV‐C irradiation. Afterward, 90 ml of distilled water was added to the bottle. The resulting mixture was shaken, and its pH level was adjusted to 8.5. The sample was placed in a water bath at a constant temperature of 55°C and oscillated at 10 min intervals. Autolysis was completed in 5 hr. The samples were heated in a boiling water bath (100°C) for 10 min for enzyme inactivation and cooled down to room temperature. The samples were refrigerated and then centrifuged for 10 min at 9,000 *g*. The supernatants and residues were stored at 4°C for subsequent experiments.

#### 2.2.3 Determination of the MW distribution of the products of shrimp‐head autolysis

The MW distribution of the products of shrimp‐head autolysis in the collected supernatant was determined through high‐performance size‐exclusion chromatography. The mobile phase was 50 mmol/L Tris‐HCl buffer solution (pH 7.2). Standard MW polypeptides and N‐Hippuryl‐His‐Leuhydrate were mixed into the solution. The sample (10 µl) was placed in a Waters‐Protein‐Pak 60 column for high‐performance liquid chromatography (HPLC) elution (Prominence LC‐20A). The flow rate of the mobile phase was set as 0.7 ml/min, and absorbance was detected at 214 nm. The hydrolysate was formulated as a solution with a soluble protein content of 5 mg/min, filtered through a 0.45‐μm Millipore filter, and subjected to HPLC under the same conditions. The flow rate of the mobile phase was set at 0.7 ml/min for the detection of absorbance at 214 nm. A near‐linear correlation between the retention time (*t*) and the log of the peptide molecular mass (*Mr*) was identified. The MW regression equation was log *Mr *= −0.2448*t* + 6.4433, and the coefficient of determination was 0.99. This equation was used to investigate the MW distribution of the products of shrimp‐head autolysis.

#### 2.2.4 Free amino acid composition analysis

Individual free amino acid was analyzed using an amino acid analyzer (L‐8500A; Hitachi Co.) equipped with a visible detector under chromatographic conditions. Briefly, 20 ml of 20% 5‐sulfosalicylic acid was added into 80 ml of the shrimp‐head autolysis product solution for deproteinization, which was maintained at 4°C for 17 hr. After deproteinization, the homogenate was centrifuged at 15,000 g for 10 min at 4°C and filtered through a 0.2‐μm filter. The free amino acid contents of the filtrate were determined with postcolumn derivatization with ninhydrin. The amino acid standard solution (AA‐S‐18; Sigma) was used for identification and quantification of free amino acids. The free amino acids were expressed as milligrams per 100 g of shrimp head (mg/100 g shrimp head).

#### 2.2.5 Kinetic model of the autolysis of shrimp head after UV‐C irradiation stress

Shrimp‐head autolysis involves the degradation of body tissue structure and the gradual dissolution of protein. The following equations were used to represent the autolysis of shrimp heads. In these equations, *Y* represents the total residual protein, *TP* represents the total dissolved crude protein, *PP* represents the dissolved pure protein, Pe represents the autolysis product with MW < 3,000 Da, and *AA* represents the free amino acids in the autolysis product.

(1) $$dY/dt=-K_{a}\left(Y-Y_{\infty }\right);$$

(2) $$d\left[\mathrm{TP}\right]/dt=-\alpha_{\mathrm{TP}}\left(dY/dt\right);$$

(3) $$d\left[\mathrm{Pe}\right]/dt=-\alpha_{\mathrm{Pe}}\left(dY/dt\right);$$

(4) $$d\left[\mathrm{AA}\right]/dt=-\alpha_{\mathrm{AA}}\left(dY/dt\right);$$

(5) $$d\left[\mathrm{Pe}\right]/dt=\beta_{\mathrm{TP}}\left(d\left[\mathrm{TP}\right]/dt\right)$$

All of the coefficients were constant. The integral initial conditions for *t* = 0 were set as follows:

$$Y=Y_{0},\left[\mathrm{TP}\right]={\left[\mathrm{TP}\right]}_{0},\left[\mathrm{Pe}\right]={\left[\mathrm{PP}\right]}_{0}$$

An integral equation can be obtained by using the following equations:

(6) $$\text{Ln}\left[\left(Y-Y_{\infty }\right)/\left(Y_{0}-Y_{\infty }\right)\right]=-K_{a}t;$$

(7) $$Y=\left(Y_{0-}Y_{\infty }\right)e^{-Kat}+Y_{\infty };$$

(8) $$\left[\mathrm{TP}\right]={\left[\mathrm{TP}\right]}_{0}-a_{\mathrm{TP}}\left(Y-Y_{0}\right);$$

(9) $$\left[\mathrm{TP}\right]=-a_{\mathrm{TP}}Y+C_{1};$$

(10) $$\left[\mathrm{Pe}\right]={\left[\mathrm{Pe}\right]}_{0}+a_{\mathrm{Pe}}\left(Y-Y_{\infty }\right);$$

(11) $$\left[\mathrm{Pe}\right]=-a_{\mathrm{Pe}}Y+C_{2};$$

(12) $$\left[\mathrm{Pe}\right]={\left[\mathrm{Pe}\right]}_{0}+\beta_{\mathrm{TP}}\left[\mathrm{TP}\right]-{\left[\mathrm{TP}\right]}_{0};$$

(13) $$\left[\mathrm{Pe}\right]=\beta_{\mathrm{TP}}\left[\mathrm{TP}\right]+C_{3};$$

(14) $$\left[\mathrm{AA}\right]=-a_{\mathrm{AA}}Y+C_{4}.$$

If *Y*_∞_ = 0, then d[*Pe*]/d*t* = *β_TP_* (d[*TP*]/d*t*) can be simplified as follows:

(15) $$Y=Y_{0}e^{-\;Kat}.$$

## 3 RESULTS AND DISCUSSION

### 3.1 Autolysis kinetic model of shrimp head after UV‐C irradiation stress

The hydrolysis kinetics model reflects the mechanisms underlying hydrolysis, and an experimental model can be established on the basis of the reaction mechanism reflected by experimental data (Qi & He, 2006). A previous study mainly focused on the hydrolysis of commercial proteases (Alpay & Uygun, 2015; Barros & Malcata, 2004; Pagán, Ibarz, Falguera, & Benítez, 2013). In addition, some researchers began to study the kinetics of enzymatic hydrolysis assisted by physical means. Maresca and Ferrari (2017) found that high hydrostatic pressure accelerated the hydrolysis of bovine serum albumin by α‐chymotrypsin and trypsin. The hydrolysis reaction followed zero‐order kinetics, and enzyme inactivation conformed to second‐order kinetics. Yu, Zeng, Zhang, Liao, and Shi (2016) reported that ultrasonic pretreatment enhanced the reaction rate of gelatin hydrolysis and that sonication changed the kinetics of gelatin hydrolysis.

**Figure 1:**
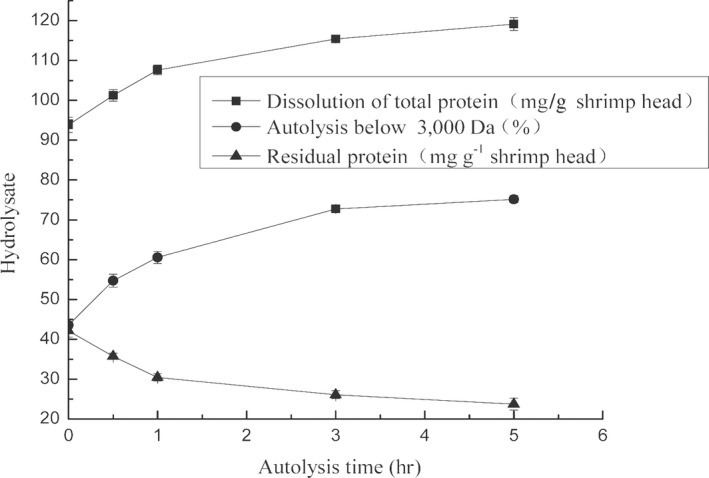
Autolysis kinetic curves of shrimp head within 5 hr after UV‐C irradiation stress

We found that the autolysis rate of shrimp heads subjected to UV irradiation was enhanced (Cao et al., 2014). In accordance with the above research results, we performed the autolysis of shrimp‐head homogenate after UV‐V irradiation stress under the following conditions: pH of 8.5, temperature of 55°C, and substrate concentration of 1:3 (W:V). We determined the total protein content in the supernatant of the autolysis product after centrifugation, the content of autolysis products with MW < 3,000 Da, and total protein content in the residue after centrifugation. Data were collected by using a minimum variance regression fit. The rate constant of shrimp‐head autolysis was 0.173, the generation coefficient of total protein dissolution was 1.5117, and the generation coefficient of autolysis products with MW < 3,000 Da was 1.769 (Figure 1 and Table 1). The kinetics model of shrimp‐head autolysis at 0–5 hr of the reaction was *Y* = 37.681e^−0.173t^, and the dissolution of the autolysis products conformed to a first‐order reaction rate equation. The kinetics model of the dissolution products exhibited exponential attenuation. Good linear correlations were observed between the dissolution products of the total protein, autolysis products with MW < 3,000 Da, and the residual total protein. The dissolution rate of autolysis products with MW < 3,000 Da increased as total protein solubility increased. These two parameters showed a good linear correlation.

**Table 1:** Fitted results of the autolysis kinetic parameters of shrimp head after UV‐C irradiation stress

Parameters	Fitted results	*R* ^2^
*K_a_* (/hr)	0.173	0.86
*α_TP _*(mg/g)	1.512	0.99
*a_Pe _*(%)	1.769	0.99
*β_TP _*(mg/g)	1.210	0.98

### 3.2 Effect of temperature on the autolysis rate of shrimp head after UV‐C irradiation stress

**Figure 2:**
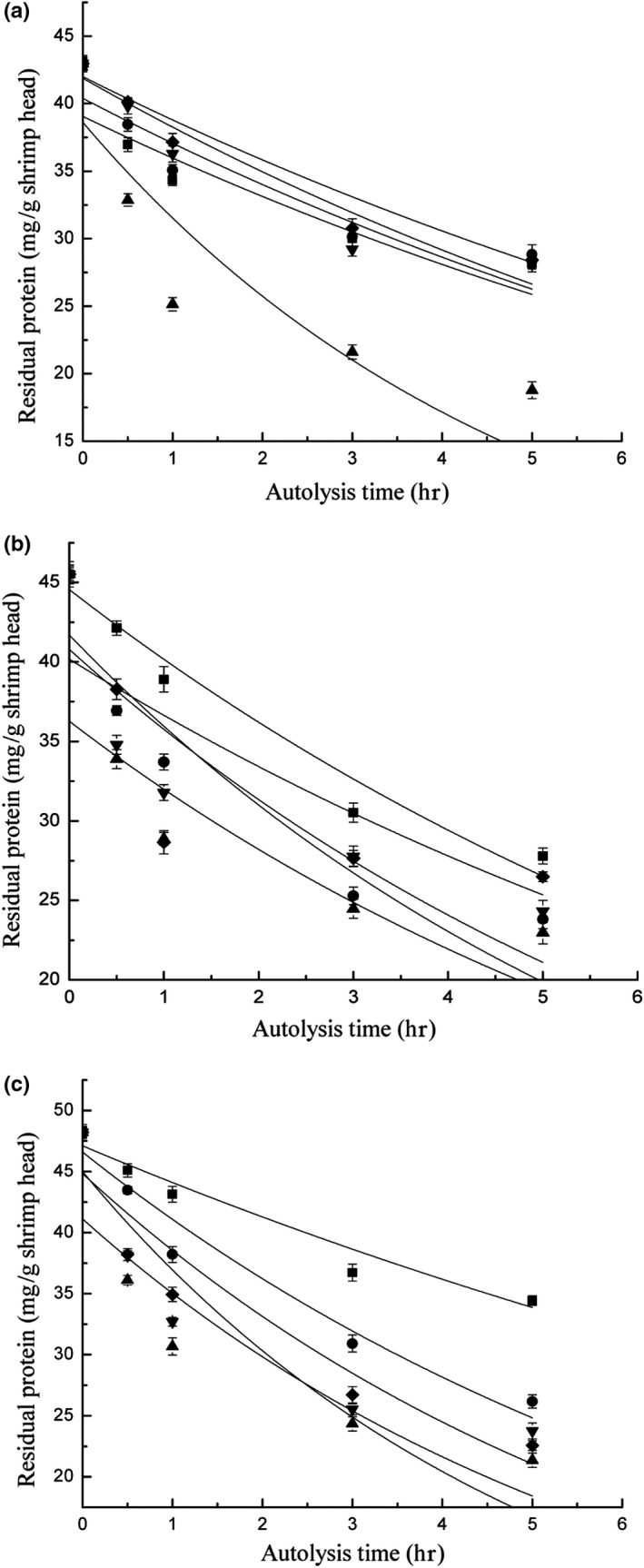
Autolysis kinetic curves of shrimp head after UV‐C irradiation stress: (a) different temperatures; (b) different pH; (c) different substrate concentration

Temperature is an important factor that influences the autolysis rate constant (Figure 2a and Table 2). At 55°C, the autolysis rate constant reached the maximum value, which was 2.17 times the value of the rate constant at 45°C. The autolysis rate constant changed as the temperature varied from 45°C to 55°C. The autolysis rate constant initially increased as temperature increased and declined gradually as the temperature further increased from 45°C to 55°C. The rate constant at 65°C was only 49% of that at 55°C. Shrimp‐head autolysis is an enzymatic reaction because endogenous enzymes in shrimp heads are activated under suitable conditions. Shrimp‐head autolysis was accelerated when the optimal temperature for the enzymatic reaction was reached. This phenomenon elevated endogenous enzymatic activity to the peak value. However, autolysis rate gradually decreased at temperatures that exceeded the optimal temperature because endogenous enzymes were deactivated. We found that shrimp heads contain various endogenous enzymes. Moreover, we found that UV‐C irradiation stress drastically enhanced the activities of these enzymes. These findings will be published later. The identified enzymes demonstrated their activities at different optimal reaction temperatures. Experimental data showed that the highest autolysis rate constant was observed at 55°C. Thus, 55°C was the optimal temperature for the activities of endogenous enzymes in shrimp heads irradiated with UV‐C.

**Table 2:** Effects of temperature, pH, and substrate concentration on the autolysis rate of shrimp head after UV‐C irradiation stress

	Temperature (ºC)				
	45	50	55	60	65
*K_a_* (/hr)	0.081	0.082	0.173	0.092	0.087
*Y*_0_(mg/g)	42.97				
*R^2^*	0.86	0.87	0.81	0.91	0.95

	pH				
	6.5	7.5	8.5	9.5	10.5
*K_a_* (/hr)	0.105	0.138	0.173	0.117	0.109
*Y_0_*(mg/g)	45.52				
*R^2^*	0.96	0.88	0.85	0.82	0.83

	Substrate concentration (W: V)				
	1:1	1:2	1:3	1:4	1:5
*K_a_* (/hr)	0.096	0.125	0.173	0.151	0.155
*Y_0_* (mg/g)	48.21				
*R^2^*	0.96	0.96	0.85	0.85	0.93

### 3.3 Effect of substrate concentration on the autolysis rate of shrimp head after UV‐C irradiation stress

Substrate concentration is another important factor that influences the autolysis rate constant. The autolysis rate constant reached the maximum value of 0.173 at 1:3 substrate concentration but was only 56% of the maximum value at the minimum substrate concentration of 1:1 (Figure 2b and Table 2). The proportion of enzymes to substrates affected the rate of the enzymatic reaction. The enzymatic reaction accelerated as the enzyme concentration increased in the presence of sufficient amounts of substrate. However, enzymatic hydrolysis decelerated as enzyme concentration decreased because substrate–enzyme binding decreased. Endogenous enzymes in the shrimp‐head homogenate can satisfy the requirements of autolysis and support soluble protein dissolution at the appropriate solid‐to‐liquid ratio because they are not excessively diluted. Thus, the rate of shrimp‐head autolysis can reach the maximum value. Barros and Malcata (2004) reported that the fastest rates of the enzymatic reactions of whey protein were obtained at a substrate concentration of 4:500 and that the rate of the enzymatic reaction declined rapidly below this ratio. Our results showed that the rate constants of shrimp‐head autolysis reached high levels at the substrate concentration of 1:3. Given the relationship between substrate concentration and autolysis rate, we identified 1:3 as the most suitable substrate concentration of endogenous enzymes in shrimp heads.

### 3.4 Effect of pH on the autolysis rate of shrimp head after UV‐C irradiation stress

Among the three factors, pH exhibited the weakest influence on the rate constant of shrimp‐head autolysis. The maximum rate constant was only 1.35 times the minimum value (Figure 2c and Table 2). However, the effect of pH on the rate constants of shrimp‐head autolysis showed a regular pattern. The rate constant reached the maximum value at pH 8.5 and either increased or decreased regularly with changes in pH. Shrimp‐head autolysis is an enzymatic reaction given that endogenous enzymes in shrimp heads are activated under certain conditions. Enzymatic activity can reach the maximum value at the optimal pH. The substrate dissociation rate was most suitable for its combination with enzymes. Endogenous enzymes in shrimp heads comprise a mixture of various enzymes, such as endogenous proteases. Each endogenous enzyme has a specific optimal pH. Endogenous enzymes have different optimal pH values given their different compositions. Therefore, we obtained dissimilar rate constants when we performed shrimp‐head autolysis under different pH conditions. In the present research, the variation in the autolysis rate constant with the change in pH was unremarkable. This result may be attributed to the high neutral and basophilic endogenous enzyme contents of shrimp heads (Benjakul & Michael, 1997; Lemos, Ezquerra, & Garcia‐Carreno, 2000; Muhlia‐Almazán, Sánchez‐Paz, Yepiz‐Plascencia, & Peregrino‐Uriarte, 2002). The rate constants that regressed at pH 7.8 and 8.5 were high. Given the relationship between pH and autolysis rate constants, the optimal pH values for the activities of endogenous enzymes in shrimp heads range from 7.5 to 8.5.

### 3.5 Validation of the kinetic models of shrimp‐head autolysis after UV‐C irradiation stress

**Figure 3:**
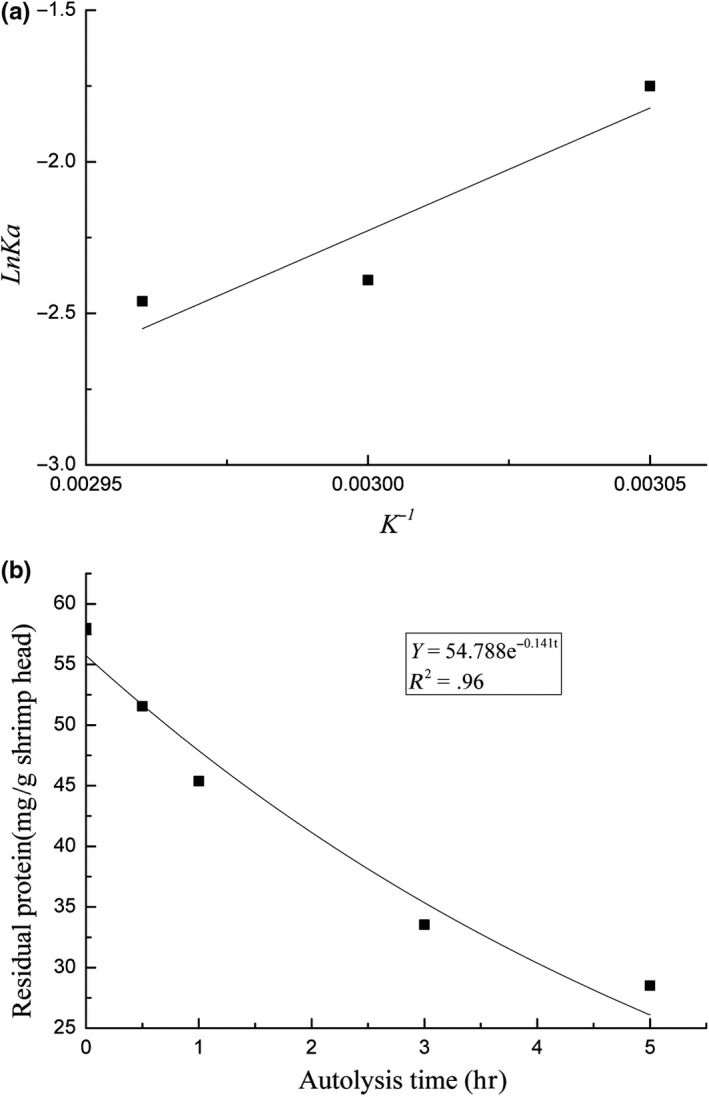
(a) Regression curve of autolysis rate constant and absolute temperature. (b) Autolysis kinetic curve of shrimp head at 57ºC after UV‐C irradiation stress

The Arrhenius equation reflects the relationship between the reaction rate constant and temperature. This relationship can be expressed as *k = Ae^−Ea/RT^* or ln*k = ‐Ea/RT + *ln*k_0_* and reflects the linear relationship between ln*k* and 1/T. The rate constants of shrimp‐head autolysis varied with temperature and reached the maximum value of 0.173 at 50°C. The autolysis rate constants gradually declined as the temperature increased from 55°C to 60°C. The order rate constant and the absolute temperature mapping (Figure 3a) coincided with the following Arrhenius equation regression: *lnKa = *8,090.2/*T‐*26.497. The equation representing the kinetics of shrimp‐head autolysis was *Y* = 54.788*e* ^−0.141t^ and was obtained at 57°C (Figure 3b). In this equation, *Ka* is 0.141. The temperature of 56.87°C was obtained after *Ka* was substituted into the Arrhenius equation ln*Ka = *8,090.2/*T‐*26.497. The similarity of this result with the preset temperature of 57°C indicates that the established kinetics model of shrimp‐head autolysis can accurately reflect the relationship between the products of shrimp‐head autolysis and residual protein content under different conditions.

### 3.6 MW distribution of the autolysis products

**Figure 4:**
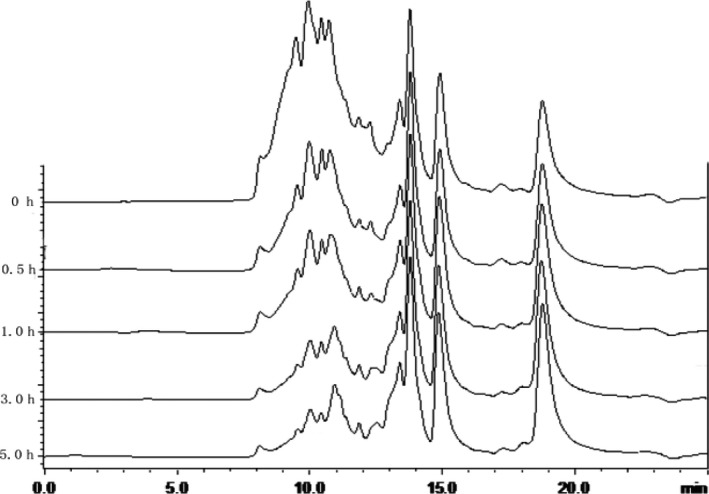
HPSEC of products during the autolysis of shrimp head after UV‐C irradiation stress

During shrimp‐head autolysis, macromolecular proteins present in shrimp heads are gradually decomposed by endogenous enzymes into peptides and free amino acids. The content of shrimp‐head autolysis products with MW < 3,000 Da reached 48% in the initial autolysis stage (Figure 4). This characteristic suggests that shrimp heads contain low‐MW peptides, free amino acids, and other low‐MW substances and that numerous soluble proteins in autolysis products are not hydrolyzed during the early stage of autolysis. Soluble protein content gradually decreased during autolysis because soluble proteins have been hydrolyzed by the UV‐C‐activated endogenous enzymes. The content of autolysis products with MW < 3,000 Da increased rapidly by 61% within 0–3 hr of shrimp‐head autolysis. The increase in the content of autolysis products with MW < 3,000 Da decelerated within 3–5 hr of autolysis because endogenous enzymes in shrimp heads were inactivated under prolonged exposure to high temperatures (55°C), and in the same time, the water‐soluble protein substrates were degraded. Shrimp‐head autolysis induced by the UV‐C‐activated endogenous enzymes ended within 5 hr. The regular pattern of MW distribution is consistent with that described in previous studies. Specifically, high‐MW substances accounted for a large proportion of products generated in the early stage of enzymatic reaction. As the enzymatic reaction progressed, the degradation of macromolecular material to small molecules accelerated, and small molecules become the dominant components. The MW distribution of the autolysate has a direct relationship with the degree of hydrolysis. The MW distribution of the autolysate can be indirectly controlled by regulating the degree of hydrolysis. Several researchers have found that the functional properties of autolysis products are related to their degree of hydrolysis (Hamada, 2000; Jamdar et al., 2010; Noman et al., 2018). Actually, the functional properties are also related to the molecular weight distribution of the autolysis products.

### 3.7 Release of free amino acids during the autolysis of shrimp head after UV‐C irradiation stress

Large protein molecules in the shrimp head are gradually degraded into small peptides and free amino acid during its autolysis. Most of the free amino acid contents increased with the prolonged autolysis time. However, the increase multiples of amino acid content were not consistent, mainly because the specific action sites of protein substrates for endogenous enzymes in shrimp head were different. The results showed that the amount of free amino acids increased remarkably and glutamic acid content was the highest. The dissolution of aliphatic amino acid was predominant during the autolysis of shrimp head, but Asp exhibited the highest increase in dissolution rate (Table 3). Furthermore, the contents Glu, Cys, Ser, Ile, and Val increased remarkably. The contents of these amino acids were 3.4–10.5 times the size of the initial content after 5 hr of autolysis. The content of heterocyclic amino acids (Trp and His) also considerably increased, and their final contents were 3–3.5 times the initial contents. Conversely, the content of aromatic amino acids increased slightly. After 5 hr of autolysis, Gly was the most abundant amino acid among the autolysis products. Arg, Leu, and Glu were also abundant. These amino acids constituted > 39% of the total amino acids. However, these results are not consistent with those of Cao et al. (2009). The main reason is that the endogenous enzymes in shrimp head underwent a certain degree of modification UV‐C irradiation stress, many previously inactive endogenous enzymes or zymogens were activated.

**Table 3:** Free amino acids released during the autolysis of shrimp head after UV‐C irradiation stress (mg/g shrimp head)

Amino acid	Time (hr)				
	0	0.5	1	3	5
Asp	0.374	0.990	1.962	3.304	3.938
Thr	2.103	3.029	4.807	5.591	5.903
Ser	0.785	1.280	1.976	2.691	2.805
Glu	0.869	1.734	3.494	6.043	6.923
Gly	6.457	6.347	8.246	9.541	9.482
Ala	2.647	3.399	4.921	6.204	6.116
Cys	0.084	0.187	0.326	0.433	0.451
Val	1.298	2.068	3.252	4.209	4.459
Met	0.920	1.327	1.734	2.112	2.141
Ile	1.126	1.863	2.805	3.351	3.931
Leu	3.326	4.367	6.088	7.344	7.740
Tyr	2.959	3.131	3.520	3.549	3.315
Phe	2.790	2.739	3.934	4.444	4.488
Lys	4.063	4.528	5.724	6.750	6.901
His	0.513	0.297	1.192	1.646	1.778
Arg	5.599	5.614	7.359	7.971	7.883
Pro	2.325	2.284	2.666	2.779	2.860
Trp	0.033	0.055	0.070	0.088	0.099

## 4 CONCLUSIONS

We investigated the principle of peptide and amino acid release during shrimp‐head autolysis after UV‐C irradiation stress. The kinetics equation of the release of nitrogen‐containing substances during 0–5 hr of shrimp‐head autolysis can be expressed by using the following first‐order reaction rate equations: *Y* = 37.681*e* ^−0.173^ *^t^*, *Pe* = −1.769*Y* + 74.156, and *TP* = −1.5117*Y* + 60.866. The autolysis rate of products with MW < 3,000 Da increased rapidly from 0 hr to 3 hr of shrimp‐head autolysis. The aliphatic amino acids showed a higher amount release than that of other amino acids during the autolysis. However, the activation mechanisms of endogenous enzymes in shrimp‐head waste after exposure to UV‐C irradiation warrant further exploration to optimize and control autolysis technology. Our research team will focus on these topics in future works.

## CONFLICTS OF INTEREST

The authors declare no conflict of interest.

## ETHICAL APPROVAL

This study does not involve any human or animal testing.
